# Co-creating Wellby—a mobile app and wearable for student well-being management guided by a needs assessment and co-design

**DOI:** 10.3389/fdgth.2025.1560541

**Published:** 2025-04-29

**Authors:** Justin Laiti, Jennifer Donnelly, Elaine Byrne, Pádraic J. Dunne

**Affiliations:** Centre for Positive Health Sciences, Royal College of Surgeons in Ireland, Dublin, Ireland

**Keywords:** student well-being, participatory design, co-design, mobile app, wearable, positive technology

## Abstract

**Background:**

Adolescents need additional well-being support, particularly in stressful periods such as during the final years of secondary school. These students are growing up in an increasingly digital world, however there is a lack of mobile applications specifically designed to support adolescent students' well-being. Because of this, there is a need for co-created digital tools that are built to promote thriving in this population. The aim of this study was to explore how digital tools, such as a mobile app and wearable, can be used to address Irish secondary school student well-being needs through a collaborative co-design process with students.

**Methods:**

Groups of students at four schools were sent a needs assessment to understand student's most pressing well-being needs. Co-design sessions were conducted with a group of students at each school, following the confirmation of stress and sleep as students' main well-being priorities and their interest in digital support tools.

**Results:**

Students' conversations and designs from these sessions helped to uncover important elements of a well-being toolkit that they named, Wellby. The Wellby toolkit is comprised of a bespoke mobile app and wearable device for use by individuals. Participating students identified requisite elements of Wellby support that included self-tracking tools, supports for stress, and customizable features.

**Discussion:**

These insights from Irish secondary school students helped to shape a student-centered well-being support tool and provide an example of co-created positive technology.

## 1 Introduction

Adolescence, defined by the United Nations as spanning ages 10 to 19, represents a formative period that can shape both immediate and long-term health outcomes for a young person (1, 2). During this time, lifestyle habits, such as diet, physical activity, and stress management, can encourage positive health later in life and reduce the burden of preventable non-communicable diseases (NCD) (2, 3). However, despite the potential for interventions to reduce health burdens in this growing population, adolescents' well-being needs are often overlooked due to relatively lower rates of chronic disease and unique barriers faced by adolescents in accessing support (4, 5). Importantly, social, environmental and demographic factors significantly impact adolescent health outcomes (6, 7). Therefore, understanding local contexts is critical for designing tailored interventions for adolescents.

There are several existing research studies investigating adolescent well-being in Ireland. The Growing Up in Ireland study highlighted the significant mental health challenges faced by adolescents in Ireland (8). This study followed over 19,000 infants and children in the Republic of Ireland over the course of 9 years and showed that 10% of adolescents received formal diagnosis for depression or anxiety with higher rates among girls (8). Additionally, only 44% of adolescents identified as needing specialized mental health services, were using them (8). In secondary school in particular, students are experiencing increased levels of loneliness and stress as they approach final year exams, and these have escalated further since the COVID-19 pandemic (9–11).

Digital interventions, particularly mobile apps, offer unique affordances for supporting adolescent well-being which are increasingly relevant given the prevalence of smartphone use among young people. Mobile apps can be scalable, accessible, and easily integrated into adolescents' daily lives, offering personalized, context-sensitive content. They provide multiple layers of support, including cognitive, emotional, and social resources, and can encourage self-awareness and behavior change through features such as self-monitoring, mood tracking, and goal-setting (12). These apps can be used alone or to complement other health and well-being interventions. For example, they can be augmented by wearable devices to provide biofeedback, or real-time monitoring of activity and physiological metrics such as heart rate variability (HRV), to help individuals make informed decisions in managing their health and well-being (13, 14). Used together, mobile apps and wearables offer a promising digital intervention for adolescent well-being. However, digital tools that address adolescent-specific needs and preferences remain underdeveloped.

Despite the promise of digital interventions to support health and well-being, there is currently a need for high-quality, psychologically-informed mobile apps focused on adolescent stress and overall well-being (15–17). Many existing mobile apps that show promising results focus on general mental health or stress in adult and young adult populations (18–20), with limited attention to the factors that influence adolescent engagement. Rather than using the same digital tools that were created for adults, there is a need to develop custom solutions for adolescents who often use these tools in unique contexts (21). A survey of adolescents in the UK found that the most commonly used mental health apps were Headspace, Calm, and Daylio Journal (22). While these apps offer features such as psychoeducation, guided relaxation exercises, and mood tracking, they are designed for the general population rather than adolescents specifically.

Of the digital interventions focused on adolescents, few incorporate meaningful collaboration with young people in their design or development. Among those that do, many are web-based platforms targeting chronic disease management or clinical mental health conditions rather than well-being (23). The ClearlyMe mobile app, developed for Australian adolescents, offers an example of a co-design process used to develop a digital cognitive behavioral therapy (CBT) intervention (24). However, the co-design of ClearlyMe began with a predefined goal of creating a CBT-based tool rather than allowing adolescents to determine what kind of intervention would best support their needs. In contrast, the development of the Niggle app involved a more open-ended co-design approach, where adolescents were invited to explore how technology could support their well-being and to guide the direction of this tool (25). Similarly, the non-profit Hopelab focuses on human-centered digital supports for young people, publishing reports such as an outline of youth's experience with social media (26) and the product design of apps to support college student mental health (27). These examples highlight the need not only for adolescent-specific digital tools, but also for participatory processes that actively involve young people to shape new digital well-being interventions.

Participatory approaches can help to ensure that health and well-being interventions are addressing relevant needs and align with the preferences of their intended end-user (28, 29). As Odoewa notes, participatory research is not new but has deep roots in global and cultural traditions (30). There is a wide range of modern frameworks including Community Based Participatory Research (31), Participatory Action Research (32), and Radical Participatory Design (30). While many design processes involve users only after a prototype has been developed, typically through feedback or usability testing, co-design offers a more inclusive approach by positioning participants as equal partners throughout the design process, from conceptualization to implementation and evaluation (33).

In this study, co-design was implemented through the lens of Radical Participatory Design (RPD), which emphasizes a shift in power dynamics, transferring decision-making authority from designers to end-users (30, 34). Unlike approaches that seek user input only after core design decisions have been made, RPD involves participants from the earliest stages, inviting them to help define the problem space and shape potential solutions. Involving students, teachers, and caregivers in this process ensures that digital tools are engaging, accessible, and tailored to the diverse preferences and well-being goals of students. This approach has also been associated with greater ownership, accessibility, and adoption of these types of interventions by young people (23, 35).

There are several important privacy and safety considerations when implementing digital tools for adolescents. In light of the growing number of digital well-being interventions, researchers have increasingly called for more rigorous evaluation of these tools to better understand their effectiveness, safety, and long-term impact on adolescent well-being (12, 36). Digital tools present several potential risks, including concerns about data privacy, inappropriate content exposure, overuse or dependence, algorithmic bias, and insufficient safeguarding mechanisms (37–39). Custom apps for young people could help to address these risks to ensure that digital interventions truly benefit young people and avoid unintended harms. Participatory design processes can help to mitigate these risks by fostering trust between adolescents, caregivers, and researchers or developers, while also ensuring safety concerns are addressed from the outset (40).

This work contributes an example of co-designing digital tools with and for adolescents to address the gap in well-being focused technology for this age group. The iterative design process began with a needs assessment of students at four secondary schools in Ireland, including two mainstream schools and two Youthreach Centers for early-mainstream school leavers. This was followed by a series of co-design sessions where students collaborated in identifying well-being needs, generating design ideas, and providing feedback on a mobile app and wearable device intended to support adolescent well-being.

The aim of this study was to explore how the well-being needs of secondary school students can be addressed through a co-design process that focused on the development of digital tools such as apps and wearables. The central research question guiding this work was: What are Irish secondary school students' well-being goals and preferences for digital tools that could support their health and well-being? Grounded in prior research and participatory design theory, this study assumes that involving students as equal partners in shaping digital technologies will lead to more relevant, acceptable, and impactful solutions that reflect their lived experiences. To our knowledge, this is the first study to engage Irish secondary school students in a co-design process to develop a mobile app and wearable device explicitly guided by adolescents' well-being goals.

## 2 Materials and methods

This study used a mixed methods approach to guide the co-creation of a digital intervention for supporting secondary school students' well-being goals, beginning with an online needs assessment and in-person co-design workshops. Quantitative data captured in the needs assessment was summarized using descriptive statistics while qualitative data captured in each phase was analyzed using thematic analysis based on Braun and Clarke's approach (41).

The process followed the values outlined by the Irish Public and Patient Involvement (PPI) Ignite consortium which were later used to evaluate the co-design process from the students' perspective (42). The Double Diamond design strategy guided each phase of the design process for the mobile app and wearable by encouraging a range of ideas and opinions before making design decisions (43). Student input was crucial throughout this process and the framework on Youth-Centered Digital Health Interventions by the World Health Organization (WHO) and Radical Participatory Design (RPD) were used to guide the structure of this project and shift the decision-making power to students across each phase of the design and development process (30, 44).

### 2.1 Participants

Through collaboration with school teachers and administrators, four schools were included in this study including two mainstream schools and two Youthreach Centers. Youthreach is an alternative education program with locations around Ireland, providing specialized education for early mainstream school leavers (45). The student survey participants at the mainstream schools (School A and B) were in their Transition Year (TY), aged 15–16. TY is an optional one-year program in mainstream education between Junior Cycle and Senior Cycle with a primary focus on developing life-skills and work experience that fall outside the standard curriculum. The survey participants at Youthreach (School C and D) were in their first or second year of the program, aged 15–19. A total of 106 students completed the needs assessment and 89 participated in the co-design sessions across the four schools (Table 1). Parents/legal guardians and teachers were included in the needs assessment following recommendations from participatory design guidelines in educational settings (46).

**Table 1:** Secondary school survey and co-design participants.

School indicator	County	Type	Survey participants	Co-design participants
School A	Kildare	All-female community school	Students: *n* = 38 Parents: *n* = 32 Teachers: *n* = 8	Students: *n* = 45
School B	Wexford	Co-education community school	Students: *n* = 22 Parents: *n* = 1	Students: *n* = 16
School C	Dublin	Co-educational Alternative education (Youthreach)	Students: *n* = 20 Parents: *n* = 6 Teachers: *n* = 2	Students: *n* = 20
School D	Dublin	Co-educational Alternative education (Youthreach)	Students: *n* = 26 Teachers: *n* = 3	Students: *n* = 8

### 2.2 Needs assessment survey

A needs assessment was conducted as the initial stage of the co-creation process with the aim of understanding students' well-being needs and assessing their interest in digital well-being interventions. As part of the assessment, three different surveys were sent through an anonymous Microsoft Form to students, their parents/legal guardians, and teachers at each school. The surveys were open for responses for four weeks and promoted by the head teacher at each school.

The student survey included 14 questions focusing on three elements of lifestyle medicine (stress management, sleep, and positive connections) along with time management. These elements were chosen based on their relevance to students in existing literature and the researchers' capacity to support these areas. Students answered initial questions about their experience with each of these areas and indicated whether they wanted support in any of these areas. Additionally, students were asked about their current engagement with well-being-related apps and wearables, their interest in using student-specific digital tools, and their initial thoughts on what these tools should include.

The brief surveys sent to teachers and parents/legal guardians asked participants to select the areas of lifestyle medicine important for their student and provided an open-response question for any concerns regarding the project. Data from the survey was summarized using descriptive statistics for quantitative questions, such as multiple choice or numerical responses, and thematic analysis for open-response questions, where key patterns and themes were identified through coding and categorization.

The survey was initially distributed to School A and B. Before sharing it with Youthreach centers (Schools C and D), the teachers reviewed the survey and made any necessary changes to gather information important to them and to ensure questions were clear for the students. Demographic information (age, race/ethnicity, and gender) was added into the survey later and collected for Youthreach students (Table 2).

**Table 2:** Youthreach student demographics.

Demographic	Student participants *n* (%)
Age	
15	3 (7%)
16	16 (35%)
17	12 (26%)
18	9 (20%)
19	5 (11%)
Not specified	1 (2%)
Race or ethnicity	
White Irish	36 (78%)
White Irish Traveller	6 (13%)
Other White/Caucasian	1 (2%)
Black or Black Irish	2 (4%)
Not specified	1 (2%)
Gender	
Woman	29 (63%)
Man	14 (30%)
Non-binary	1 (2%)
Not specified	2 (4%)

### 2.3 Co-design sessions

A series of co-design sessions were conducted as a follow up to the needs assessment to create a space for students to take the lead in defining the focus of the digital intervention in this study. Each session was 1 to 1.5 h and followed a consistent structure where two of the authors (JL, JD) briefly presented information relevant to the session topic, followed by student-led discussion groups, and finally a student design-based activity (Table 3). The session topics were selected as a starting point for the students to contribute specific ideas on functional and aesthetic aspects of the app and wearable. By introducing the topics of Positive Health Sciences (PHS) and positive technologies, we aimed to help students align their ideas with their well-being. PHS provides frameworks that integrate positive psychology, lifestyle medicine, and behavior change science to promote human flourishing (47). Positive technology builds upon this by applying these principles to the design, development, and implementation of digital tools that enhance emotional experience and meet people's psychological needs (48, 49). Each school went through all the sessions except School D which was added later in the study and completed one condensed co-design session which focused on the discussions and activities in the first two sessions outlined in Table 3.

**Table 3:** Co-design session overview.

Session sections	Session number			
	1	2	3	4
Presentation Topic	Co-design outline, description of Positive Health Sciences (PHS)	App design, positive technologies	Evidence-based health-related educational content	Wearable devices, Heart Rate Variability (HRV)
Discussion Topic	Well-being survey results discussion	Positive technology app features	Digital wellness resources	Wearable device components and 3D printing
Activity Overview	Paired students created ideas for a technology they would create to support student well-being and pitched it to the rest of the group	Paired students designed wireframes, using paper or the program Miro, for an app feature that they discussed	Students designed an evidence-based educational resource related to one of their well-being goals	Students added comments to wireframes (layouts) in Miro and voted on which discussed features they would use

Multiple sources of data, including surveys, focus groups, and hands-on design-based activities, were integrated into the co-design process to provide diverse opportunities for students to share insights into their needs and opinions (24). One design activity focused on creating app wireframes which represent the basic layout and functionality of an app. A designated student note-taker recorded and summarised any points raised in discussion groups. Focus group discussion notes and student-created designs were analysed using thematic analysis and content analysis, respectively. These were coded and categorized to identify students' preferences related to the apps' functionality and aesthetics. Students also voted on the final features to be included in the design of the app at the end of the co-design.

Additionally, Positive Health Sciences (PHS) and positive technology were fundamental backbones of the co-design, resulting in a focus on student flourishing instead of solely on the challenges which students faced related to their well-being (47–49). The questions and activities in each session adopted these approaches by encouraging students to consider how the proposed technology could help them achieve their well-being-related goals. In the development of Wellby, the Motivation, Engagement and Thriving in User Experience (METUX) model is being followed to ensure a well-being informed design process (50).

### 2.4 Ethical considerations

This project was conducted following the approval granted by the RCSI Research Ethics Committee (ID: 202305029, 202305030). Informed consent forms were distributed by the year head teacher at each school for the students' parent/legal guardian to sign ahead of their participation in the study. An informational video was shared with teachers and students about the project as an additional format to learn about the project and make the consent process more accessible to students. The survey included a brief description of the project and the students' participant rights. Students were also reminded of their rights as participants at the beginning of the co-design sessions. Students were made aware that their participation was voluntary, that they could leave at any time during the research and that their participation or not participation would not affect any other aspect of their schoolwork or performance. Additionally, ethical considerations for designing student-centered digital technology were discussed as part of the co-design process and outlined further in the results.

## 3 Results

This section first outlines the results from each section of the needs assessment, and then the findings from the co-design sessions and reflection.

### 3.1 Student well-being needs assessment

Across the needs assessments, stress management and sleep were the highest requested area for support, with stress being the highest at school A, B and C, tying with sleep among the School C respondents and eating well among teachers (Tables 4, 5). Additionally, when asked how stressed students feel preparing for class tasks and exams, 68%, 33%, 35%, and 33% of students indicated feeling stressed “all the time” at schools A, B, C, and D, respectively. Despite a high percentage of students feeling stressed “all the time” at Youthreach Centers (School C and D), the percentage of students seeking support for stress, in addition to the other areas, were the lowest compared to other schools (Table 4).

**Table 4:** Student well-being support responses.

Would you like support with:	Response options	School A (*n* = 38)	School B (*n* = 22)	School C (*n* = 20)	School D (*n* = 26)
Stress Management	Yes	**95%**	**86%**	**50%**	48%
	No	2%	0%	30%	28%
	Not sure	3%	14%	20%	24%
Study Habits	Yes	79%	82%	35%	28%
	No	3%	0%	30%	52%
	Not sure	18%	14%	30%	20%
Sleep	Yes	77%	62%	**50%**	**52%**
	No	5%	14%	30%	44%
	Not sure	18%	24%	20%	4%
Communication with family and friends	Yes	42%	32%	30%	24%
	No	24%	36%	55%	76%
	Not sure	34%	32%	15%	0%

**Table 5:** Teacher and parent/legal guardian on student well-being supports.

Are there any aspects of student well-being you would like us to focus on supporting?	Parents/legal guardians (*n* = 33)	Teachers (*n* = 8)
Stress	**64%**	**63%**
Eating well	48%	**63%**
Communicating with others	45%	38%
Sleep	36%	50%
Daily activity	33%	50%
Avoiding risky substances	21%	50%
Other (Social media usage)	0%	13%

### 3.2 The impact of student stress

Five themes emerged from student input on how stress influences their health: (1) sleep, (2) mental health, (3) physical health, (4) social connections, and (5) behavioral changes.

#### 3.2.1 Sleep and mental health

Students at each school noted the impact that stress has on their sleep quality, which has a strong link to poor mental health outcomes, such as feelings of anxiety or emotional instability, which may also further inhibit students sleep quality.

“It can make me more anxious and tired” (B16)

“It makes me feel rubbish during the day and … I get annoyed and upset” (C13)

Some students reported that stress causes anxiety, making it difficult to sleep and leading to more frequent fatigue.

“It can be hard to concentrate and sometimes I get minor headaches. It affects my sleep schedule and I can get very anxious”. (A15)

#### 3.2.2 Physical health

Students also noted that stress manifests in their physical health through reactions in their skin, headaches, or with stomach problems.

“It feels like you're going to faint sweaty palms really bad sore head” (C3)

“It feels like it paralyses my whole body” (A27)

“It impacts your skin” (B21)

#### 3.2.3 Social connections and behavioral changes

Students described how stress affects their relationships and behaviors.

“It makes me agitated and can cause problems amongst family members and friends” (B8)

Stress related to school tasks, like exams or homework, was reported to impact their physical health and reduce participation in activities.

“It makes you panic and lose the ability to focus and listen” (A4)

Many noted that stress leads to low moods, mood confusion, and strained social connections, sometimes causing them to withdraw from others.

“I sometimes feel stuck and go to my room and I feel stuck and I don’t know where my emotions are”. (C13)

Behavioral changes included reduced focus in school, heightened anxiety about attendance, and, for some, an increased inclination toward risky behaviors like smoking.

“It influences me to smoke hash and vapes” (C15)

### 3.3 Stress management techniques

When students were asked how they manage their stress, several themes emerged parallel to the well-being impacts outlined previously, themes were: (1) physical activity, (2) creative/emotional expression, (3) relaxation techniques, (4) social support, (5) professional support, and (6) avoidance/distractions.

#### 3.3.1 Physical activity

Numerous students described physical activities that help them work through stress including walking, playing sports, or going to the gym. A student at School B described how “*[they] like balling to relieve stress*” (B7).

#### 3.3.2 Creative and relaxation techniques

Some students also describe how journaling can help them work through their emotions. A student at School A explained how they “*like to write about how [they’re] feeling in [their] journal*”. The same student described that they like to “*read a new book, watch a movie/show, cook a meal or do some breathing exercises to calm down*” (A14). Other students noted forms of expression through painting, coloring, or doing art in general. Students shared several relaxation techniques that they use to relieve stress. Listening to music was the most common method to relax followed by watching TV or movies and practicing meditation or breathing exercises.

#### 3.3.3 Social support

Connections with both friends and family are another important component of stress management for students. One student explained how they “*talk to [their] friends about why [they’re] stressed” to help them work through their feelings* (A1). In addition to support from their direct community, students share that they seek professional support from their doctors for medications or therapy.

#### 3.3.4 Avoidance

Students at each school explained that they often try to avoid stress through distractions or by simply enduring it. One student wrote that they “*generally just pull through the issue*” (B2) while another said that they “*use [their] phone as an escape*” (B8). Another student shared that they “*go on [their] phone and listen to music to distract [themselves]*” (A3). An additional student explained that they typically find themselves “*fighting, smoking, or distancing [themselves] from everyone*” to avoid feeling stressed (C11).

### 3.4 Student sleep habits

While stress management was *a priori*ty for students, teachers, and parents/legal guardians, sleep was another major concern highlighted in the needs assessments by all groups. When students were asked how often they typically meet the recommended sleep each night for their age group (8–10 h) 18%, 15%, 5%, and 15% of students indicated meeting these 7 days a week at School A, B, C, and D respectively (51). Similarly, students indicated how often they feel well-rested. Students at both Youthreach Centers showed the highest percent of students who “rarely” feel well-rested, with 50% at School D and 28% at School C, compared to 26% and 18% at schools A and B, respectively.

### 3.5 Digital well-being interventions

The second section of the needs assessment focused on gauging participant opinions, interests, and ideas related to using digital tools, specifically mobile apps and wearable devices, to support students' well-being needs.

#### 3.5.1 Apps used for well-being support

The apps that students noted for supporting their well-being were grouped into four categories: (1) social media, (2) entertainment, (3) health tracking, and (4) mindfulness apps.

Many students noted that they use social media apps such as TikTok, Snapchat, or Instagram to support their well-being. Students at each school also included music apps like Spotify or video-sharing platforms such as YouTube. Health tracking apps were another common response, ranging from built-in health tracking apps like Apple's Health app or apps connected to wearable devices like ones manufactured by Fitbit. Additionally, some students noted mindfulness and meditation apps, including Calm, Headspace and Fabulous, that specifically support stress or anxiety management.

#### 3.5.2 Wearable device use and interest

When asked about students' current use of wearable devices (e.g., Fitbit, Garmin, Apple Watch, etc.), 61%, 29%, 5%, and 11% of students at School A, B, C, and D respectively, currently use some type of wearable health tracker.

Most students (over 60%) at each school indicated that they would be interested in using a bespoke well-being app and wearable device, specifically geared towards students of their age. There was slightly more interest in using a wearable device across the four schools compared to a mobile app.

### 3.6 Wearable feature ideation

Students also shared ideas for features of a wearable device that supports student well-being, highlighting four themes: (1) stress monitoring, (2) health metric tracking, (3) social connections, and (4) customizability and accessibility.

#### 3.6.1 Stress monitoring

Students shared that a device could be useful in helping them manage stress or calm down. They had ideas for devices that would notify them when they are feeling stressed or guide them through breathing exercises to help them relax.

“I would like it to notify me when I'm starting to get stressed” (C13)

“Like if you are having a panic attack that the device would notice that and offer ways that people cope with what is happening to them. I don’t know how they would do that, but I feel like that would be very helpful.” (A24)

“Heartbeat monitor, saying when you feel stressed and giving advice when they do” (C14)

#### 3.6.2 Health metrics and social connection

Other students shared their interest in a device that helps with sleep, activity, steps, or heart activity monitoring. One student shared that they “*would like to see [their] sleep, [their] calories burned, [their] steps, [their] heart rate*” in addition to a stress monitor (C13). Some students also wrote about social features like “*a way of communicating to friends*” (A17).

#### 3.6.3 Customizability and accessibility

Multiple students mentioned customizable features that would allow them to pick visual elements that they wanted like wallpapers or strap colours, while other students noted the importance of having accessible features for students with dyslexia or other learning differences.

### 3.7 Feedback from parent/legal guardians

Overall, parents/legal guardians were interested in supporting their student being involved in this research, noting the need for more supports for students. One parent/legal guardian shared that they needed more information on the project. Other parents/legal guardians shared that they thought this needs assessment was important to better understand the type of support that students need.

“I think this survey is important we need to know and understand how teenagers are feeling and coping”. (A5)

“I think it's a good idea, any advice and help that can be given to teenagers during this period in their lives is great”. (B1)

### 3.8 Feedback from teachers

In the survey sent to teachers, there was feedback around promoting healthy technology use for students. One teacher expressed concern about digital well-being interventions further encouraging students' reliance on their phones:

“I would be concerned that we are giving students yet another reason to be on their phones. To allow their well-being to be aligned to and managed by their phones might encourage unhealthy reliance on their phone”. (A4)

Through building relationships with teachers at each school to initiate this needs assessment, teachers also shared feedback outside of the survey. Teachers at each school noted that they could also benefit from additional well-being support and that they would like to be included alongside students in developing and testing these digital tools. These teachers also shared their own perceptions on the increasing stress and anxiety in their students, expressing a need for new methods to support their students.

### 3.9 Co-design student well-being needs reflection

The co-design aimed to build on feedback from the needs assessment and first focused on confirming students’ needs through student-led discussions of the needs assessment results and hands-on design activities. The needs assessment results were collated into graphs and themes specific to each school. Students discussed questions in groups while reflecting on these results.

#### 3.9.1 Stress management and sleep

Students largely agreed that stress and sleep management were top well-being priorities. They reflected on the needs assessment results, noting how “*students are stressed and anxious…the survey responses do support this*” (D) and that “*(students) should get more sleep*” (C). An additional theme across schools was “digital well-being”, referring to students' reflections on their relationship with technology, highlighting both the challenges and benefits of phone use for mental health.

#### 3.9.2 Digital well-being

In discussing digital well-being, students expressed mixed feelings about the role of technology in their lives and raised ethical concerns related to these tools. They noted that apps such as social media can act as both a distraction and a tool for connection depending on how they are used. Several students noted challenges with reliance on their phones, one student describing how social media “*has a way of keeping you there*” (A). Another student highlighted how apps like TikTok are “g*reat as a distraction to get you mind off things*” (D). Other students disagreed saying that TikTok was too addictive, and they preferred longer videos on platforms like YouTube (B), which another student noted is good “*for learning and getting better at their hobbies*” (D). Students also discussed that “*a lot of (students) have body issues because of*” social media because it can be difficult with comparisons and “*beauty brands*” (A). Aside from the comparison, many students noted that social media is beneficial for connecting with friends. One student explained that “*Snapchat is nice to use to talk with friends or AI*” (D). This student described how they often use Snapchat AI to talk through personal challenges which they feel more comfortable disclosing to the AI-enabled chat instead of a person who might tell someone else. Still, many students expressed concern around the emerging use of AI, explaining that “*it's too widely accessible*” and “*people use it for the wrong reasons*” (D) like creating false content.

#### 3.9.3 School-specific well-being needs

While stress, sleep, and digital well-being were common themes across each school, the feedback with each cohort also uncovered school-specific needs. The need to support student hydration was a strong theme that uniquely emerged from discussions at School A. Additionally, the source of students' stress differed, in general, between the mainstream public schools (School A & B) and Youthreach Centers (Schools C & D). One theme from School A was that students wanted help with developing timetables and study plans, expressing that much of their stressors related to school exams and assignments. However, students at Youthreach shared that, because of their less exam-focused curriculum, they don't feel as much stress due to exams, though one Youthreach student noted that “a *lot of young lads are stressed but kind of hide it*” (School C). Well-being needs noted by students at School C related to issues with sleep, finances and complications with family or other relationships. Students at School C and D expressed a need for support with navigating low moods and harmful substance use.

### 3.10 Co-design technology ideation

The student well-being needs were further explored in a design activity where students outlined ideas for inventions that would address student needs. They focused their technologies on addressing stress, sleep, time management, digital well-being, eating well and fostering relationships. The importance of simple, easy-to-use, and customizable technologies for students was emphasized by design descriptions.

“Wearable device with no distraction (no scroll)” (A)

“A device that comes in any shape, form, size, colour, design” (B)

“A convenient and simple design” (C)

Additionally, one student emphasized their interest in a student-centered wearable device.

“A lot of people have things like Fitbits, but it’d be nice to have something that's my own that I designed. I don’t currently wear one, but I’d like to”. (A)

**Figure 1:**
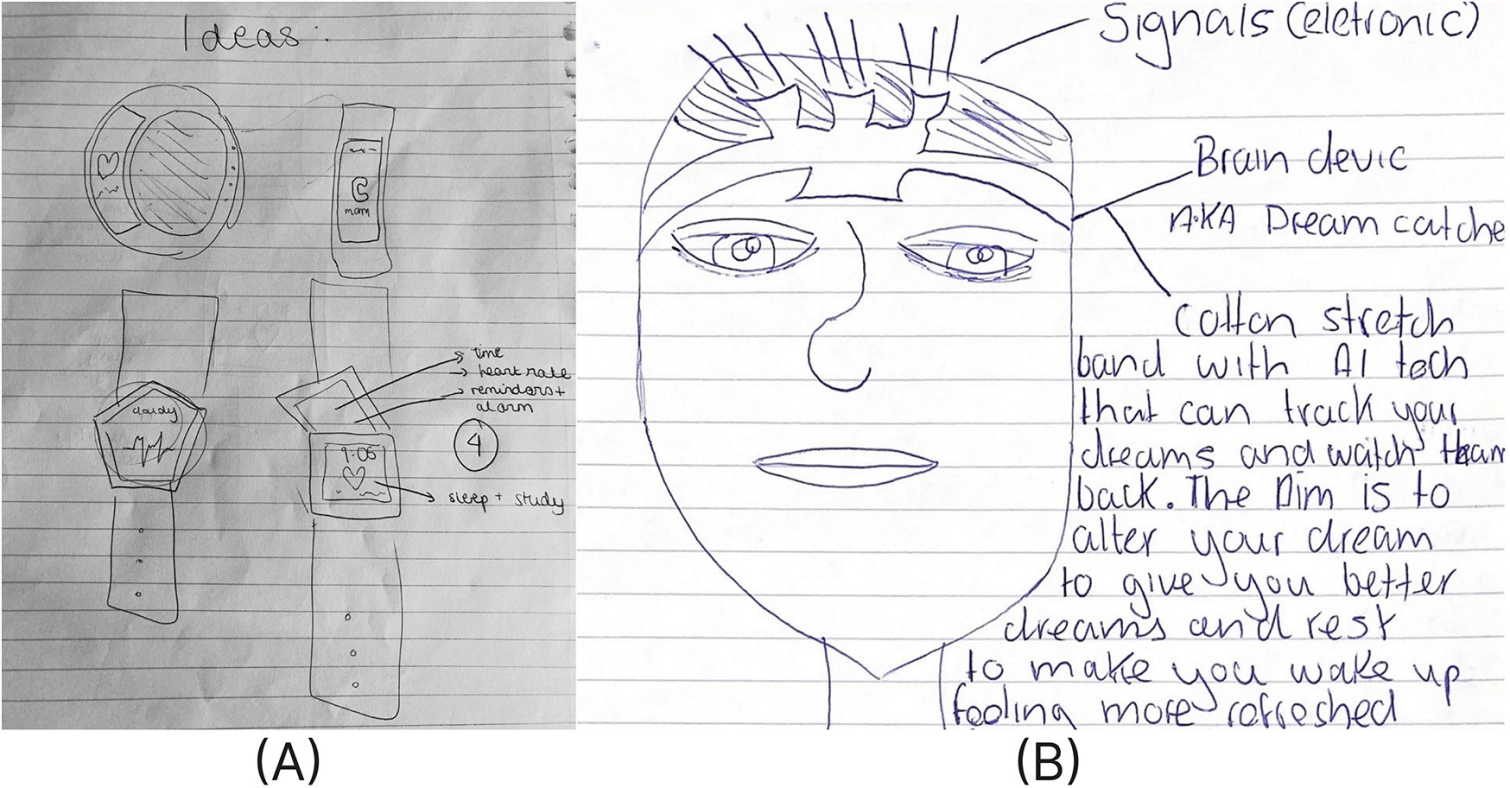
Student designs for **(A)** a wristwatch for monitoring heart activity and sleep (school A) and **(B)** a headband to track brain activity and encourage positive dreams to promote well-being (school B).

Figure 1 represents two examples of student inventions ideas including a wristwatch for sleep and heart activity tracking and a headband for controlling dreams to enhance sleep quality.

### 3.11 App features to promote flourishing

This section outlines the app features which students suggested during the co-design sessions. These were either drawn from existing apps or ones they would like to see and were intended to connect to PHS through the PERMA framework: Positive Emotions, Engagement, Relationships, Meaning, and Accomplishment (52). Each category represents an aspect of flourishing that the students felt the app should support, based on their well-being priorities. The quotes are drawn from students across the different schools with examples intended to represent the range of ideas rather than patterns specific to individual schools.

#### 3.11.1 Positive emotions

To support positive emotions, students proposed features that were encouraging like daily affirmations, inspirational quotes, and gratitude prompts. One group proposed a “*gratitude garden*” where students could “*list things they are grateful for every day and these turn into flowers in a garden*” as a visual representation and feedback of positive gratitude habit creation (A).

One student explained that having “*icons that said “good job*”” would provide a boost when completing tasks (A), while another suggested a “*daily quote*” or “*affirmation*” feature, emphasizing that seeing uplifting messages could “*make you feel a bit bette*r” throughout the day (A).

#### 3.11.2 Engagement

Students highlighted the importance of balancing screentime and promoting useful engagement through features like screen time limits, reminders to take a break and “*shortcuts, notifications or ways to access information without getting sidetracked by too many steps*” (B). One student described a notification control setting where “*students would be able to control when they get different reminders so that they aren’t at inconvenient or annoying times*” (A).

#### 3.11.3 Relationships

Students expressed a desire for app features that support healthy relationships, both by connecting with friends and accessing safe, judgment-free support. Suggested features included a secure chat function and a “*secure AI chat buddy*” (D). Students discussed how “*an AI for advice*” (D) could be helpful, while others emphasized that being able to “*talk to your friends online*” (A) would allow them to connect with peers they don't see regularly.

#### 3.11.4 Meaning

To cultivate meaning, students identified features that help them learn or engage in activities based on their interests. One student explained how an “*explore page*” could help them “*see what friend/family are up to*” and find local activities to join (C). Another student noted a feature for seeing “*famous/successful people in [their] field with ADHD or autism*” to “*provide examples of influential figures with disabilities in a certain field of interest*”, highlighting the importance of representative role models for students (D).

#### 3.11.5 Accomplishment

Accomplishment-related features aimed to help students track their progress and celebrate achievements. They proposed tools like to-do lists, goal reminders, and wellness trackers that would show progress over time, promoting both academic and personal growth. One student mentioned the benefit of a to-do list for managing tasks, while others suggested step goals or wellness reminders, such as notifications to drink water, to help them feel accomplished in self-care (A).

### 3.12 Student created app wireframes

Students designed app layouts inspired by the positive app features that they brainstormed using the PERMA model. These designs emphasized themes of goal setting and tracking, emotional support, connection, educational resources, and journaling.

#### 3.12.1 Positive emotion and accomplishment wireframes

**Figure 2:**
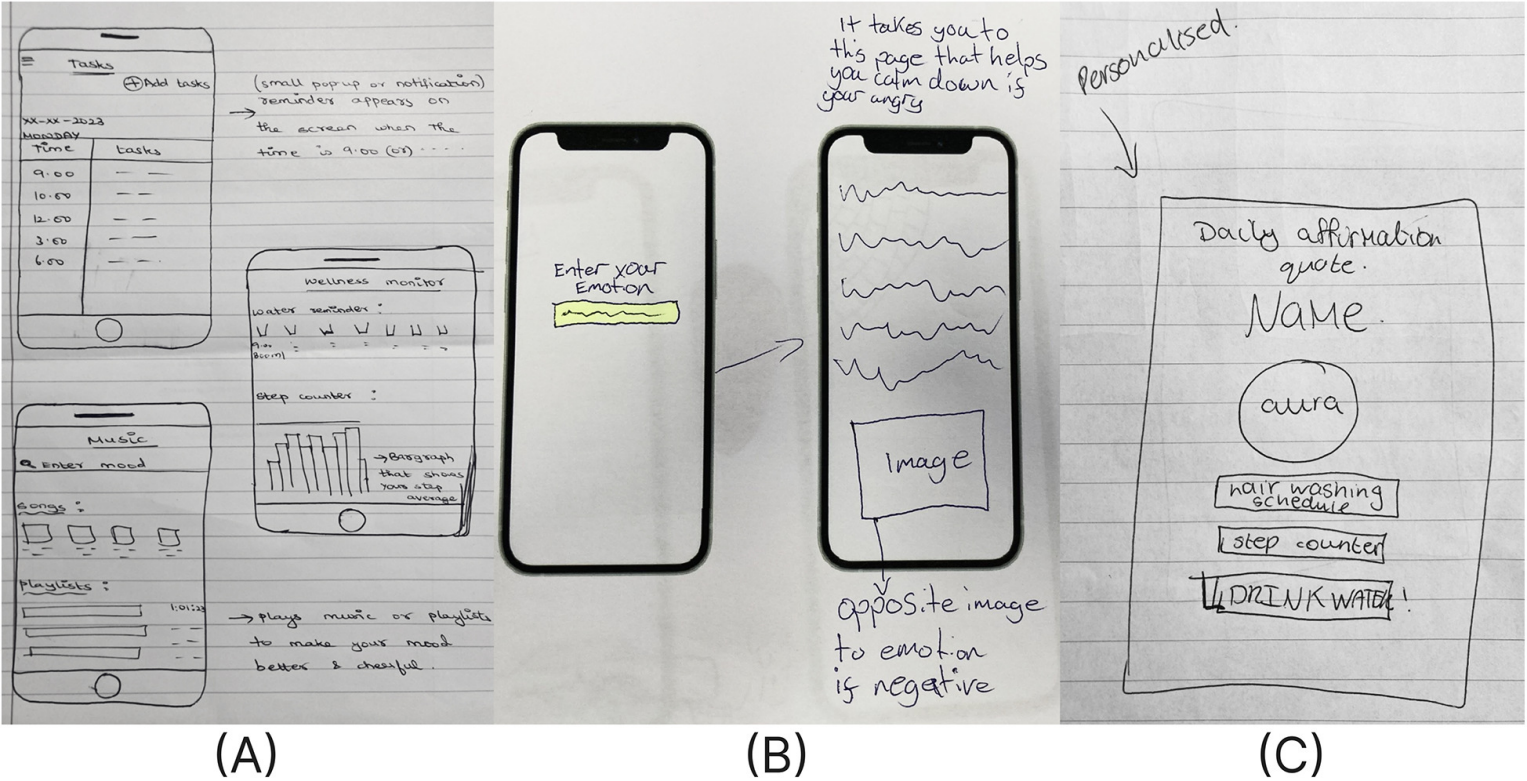
Positive emotion and accomplishment app layout designs by students for **(A)** a habit tracker for daily tasks and activity (school A), **(B)** a feature for emotion-based advice (school C), and **(C)** a screen with a daily affirmation, quote, hair washing schedule, and habit reminder (school A).

The most prevalent theme in the wireframes was goal setting and tracking, a feature strongly tied to accomplishment within the PERMA framework. Students proposed tools like calendar-based task lists, activity graphs, heart rate visualization, and reminders. These were often paired with visual feedback, such as metrics for steps or hydration levels, to reinforce habit formation. Figure 2A shows an example of a wireframe with task, hydration, and step tracking. Another recurring feature addressed emotional support, aligning with the positive emotion domain of PERMA. Students designed mood check-ins, emotion-specific advice, and music tailored to their mood. Figure 2A includes mood specific music selection while Figure 2B outlines resources based on an entered emotion. Affirmations and inspirational quotes, shown in Figure 2C, were frequently included to foster positivity and motivation.

#### 3.12.2 Engagement and relationships wireframes

**Figure 3:**
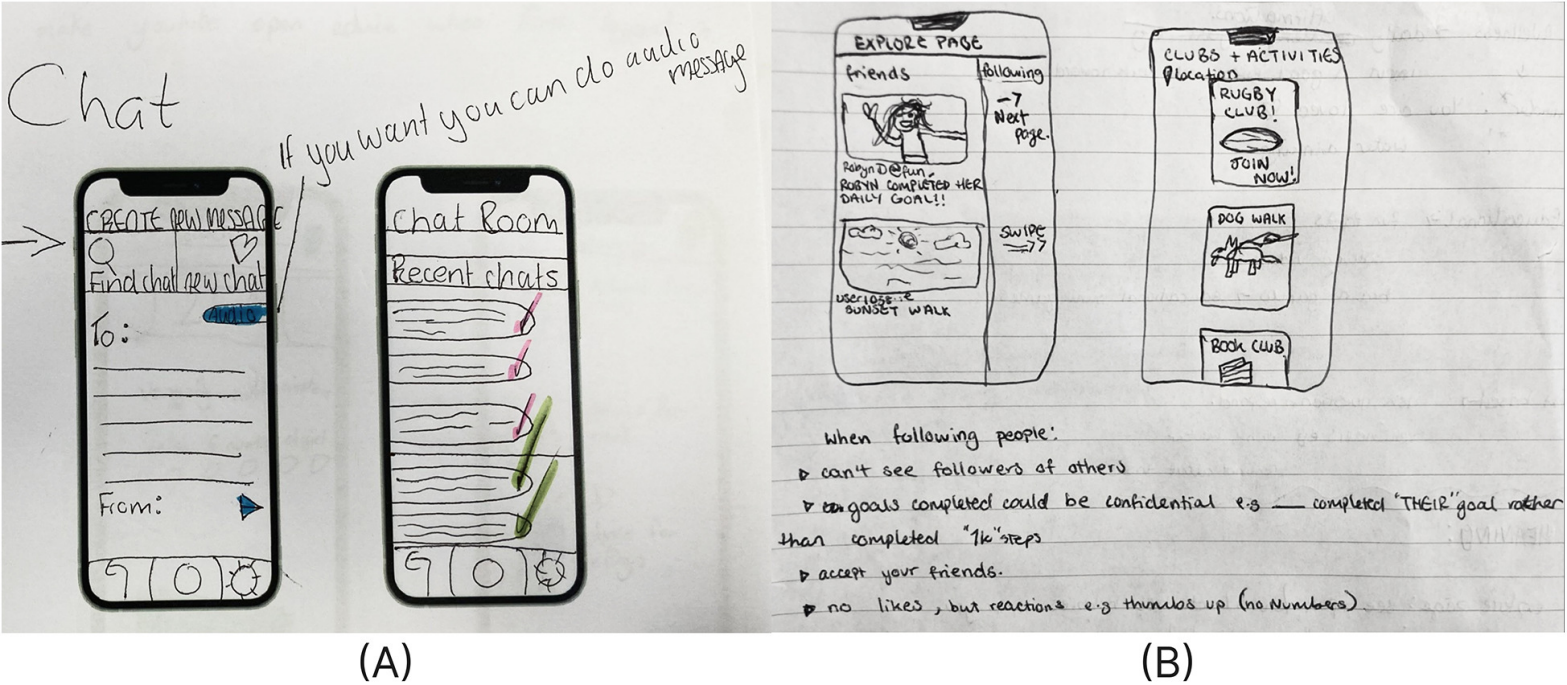
Engagement and relationship app layout designs by students for **(A)** a chat screen and chat room (school C) and **(B)** a feature for seeing friends achieving their goals and searching for activity to join (school B).

Connection emerged as another key theme in the designs, directly linking to engagement and relationships within the PERMA framework. These included ways to find activities aligned with students’ interests and connect with friends in a meaningful way. While some included chat screens like the wireframes in Figure 3A, other wireframes emphasized innovative and mindful approaches for fostering connecting with friends on an app. For example, Figure 3B illustrates a screen that displays friends' completed goals, but it specifies that people using the app “can’t see followers of others”, can keep their goals confidential, must “accept [their] friends”, and that there are “no likes”. Students designed other app features to find activities that align with their interests.

#### 3.12.3 Meaning wireframes

**Figure 4:**
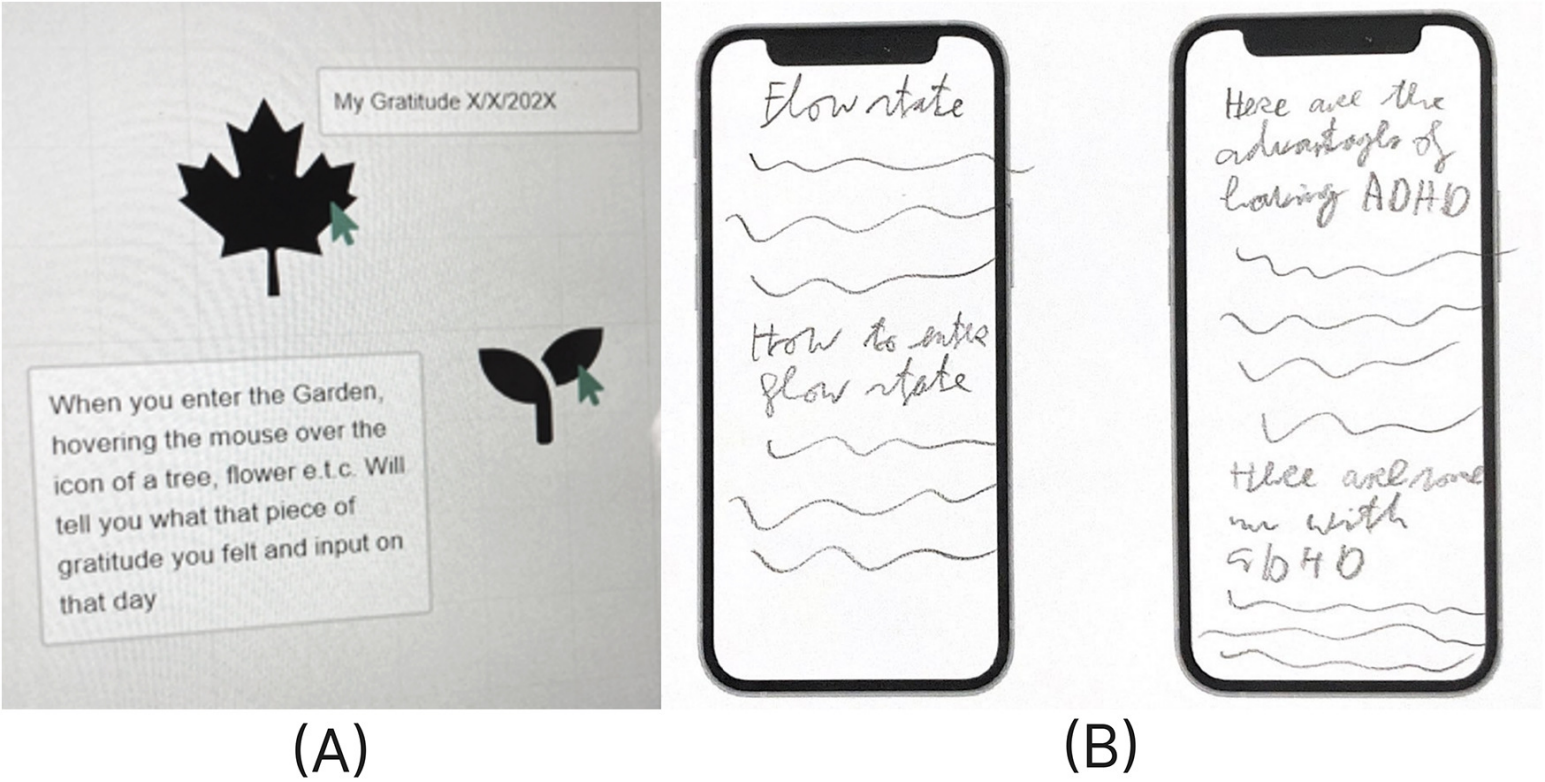
Meaning app layout designs by students for **(A)** a clickable gratitude journal with plants representing entries (school A) and **(B)** a feature with educational resources related to flow state and ADHD (school D).

Students also outlined wireframes that connected to the meaning domain of PERMA such as designs for journals or educational resources. The wireframe in Figure 4A focuses on a gratitude journal which includes a garden where plants are added based on each gratitude entry. Figure 4B presents educational resources on topics such as “flow state” and the “advantages of having ADHD”, aimed at promoting self-awareness and personal growth.

### 3.13 Initial design feedback

**Figure 5:**
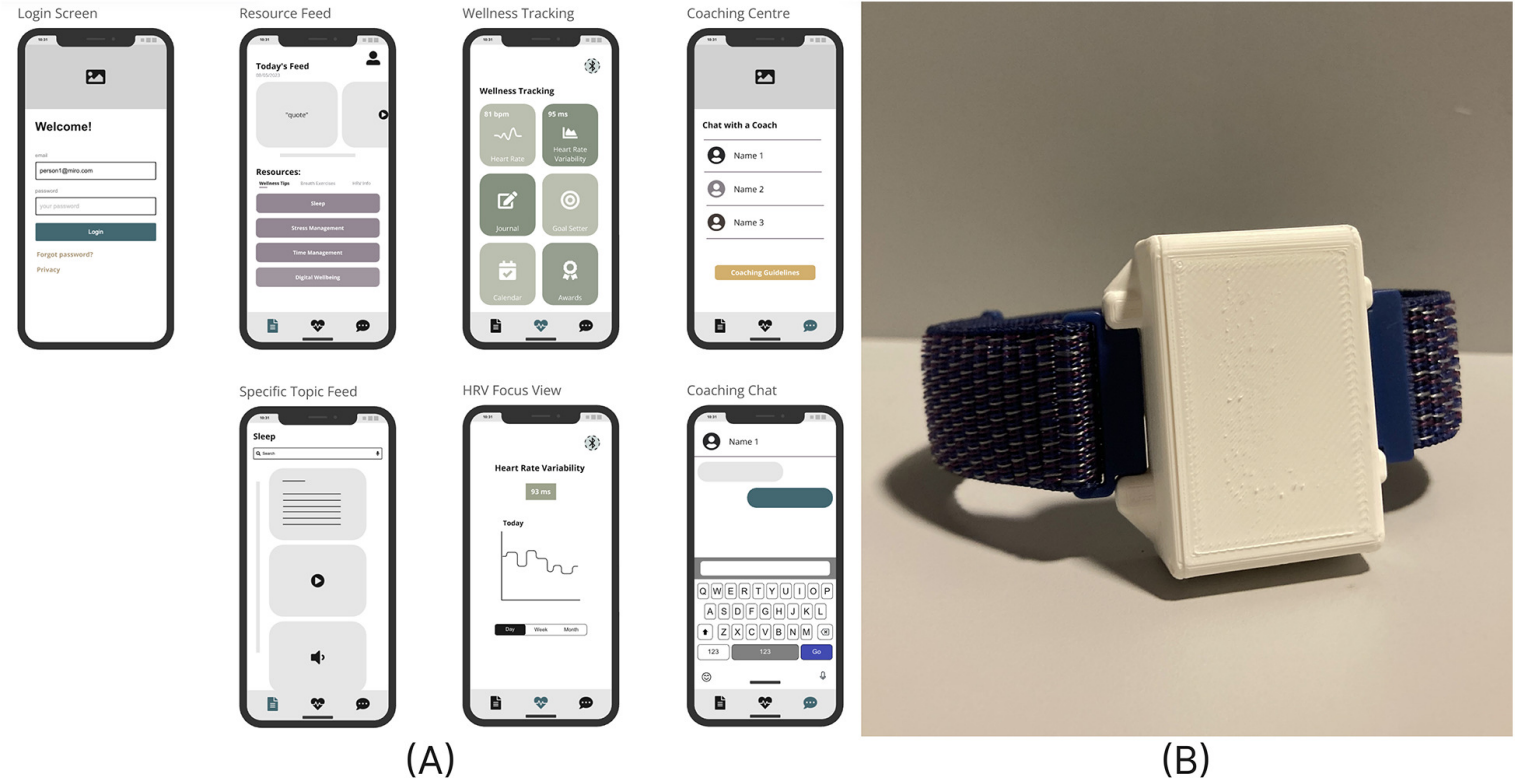
**(A)** Initial app wireframes detailing four distinct tabs and **(B)** a wrist-worn wearable heart activity monitor.

As part of the iterative design process, students were shown the initial design for both the app and wearable (Figure 5). Students were asked for their feedback on different components of these designs.

#### 3.13.1 App wireframe feedback

Based on the wireframes shown in the online program Miro, students gave feedback on the app themes, features, and the overall app-flow.

When asked about an app theme, the responses were overwhelmingly nature related. For example, students suggested “*something serene like a landscape*” (A1) and “*wildlife, rain forest, waves/beaches*” (B4). They also emphasized the importance of customizability for the theme and colors in the app: “*I think the theme should be optional for all users*” (C3).

In feedback about the features on the app, most students requested a greeting or positive quote on the app's home screen. The most popular additional features suggested for the app were a calendar with to-do lists and a goal tracker.

Regarding students' format for educational resources on the app, there was not a consistent format that students preferred given the categories: text, image, short video and long video.

Additionally, students indicated, particularly in Youthreach centers, that they do not have access to their phones throughout the school day, so they would not be able to message health coaches during the school day.

Finally, students shared their satisfaction with the overall layout of the app.

“It looks easy to use” (B10)

“I really like it! It's very accessible, and easy to navigate” (A1)

#### 3.13.2 Wearable feedback

**Figure 6:**
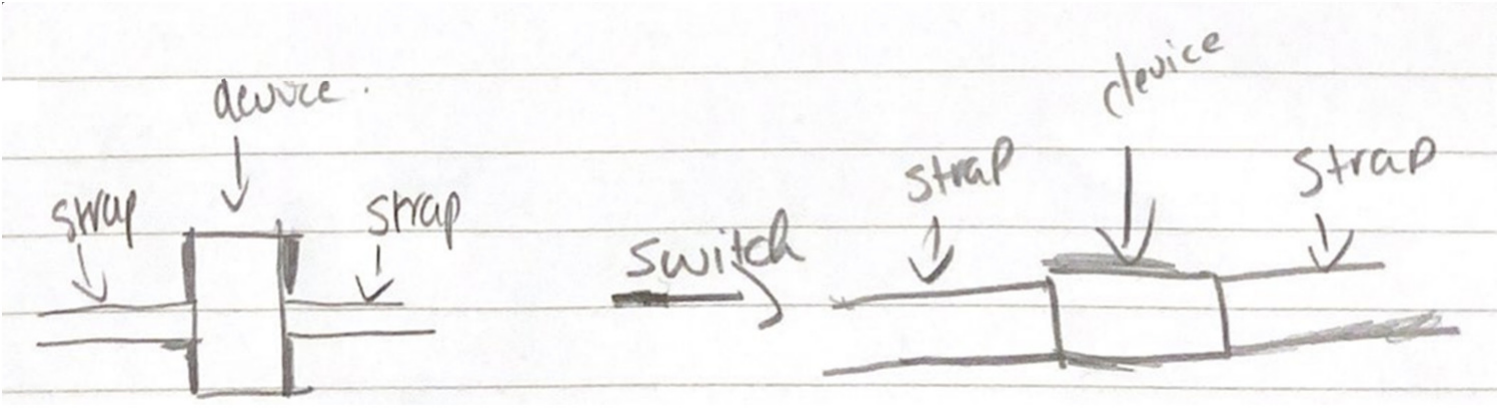
Wearable orientation changes recommendation from a group of students (school A).

Students commented on the importance of the appearance of the device. They suggested changes such as making it smaller, more discrete, sleek, rounding the corners, and changing the orientation of the first prototype. Students were also invited to draw their ideas for changes such as the orientation change in Figure 6.

“Smaller and slimmer device and band” (C3)

“Make it more round, streamlined, not boxy” (C5)

“Casing seems a bit bulky, may get in the way or caught in clothes” (A20)

Many students commented on the ability to customize the device in various ways such as choosing the case and strap color or material.

“Change of colour and design like silk or fluffy material” (C2)

“Beaded strap, looks like jewelry? Cloth straps are annoying when wet” (A3)

“Can we choose the color of the casing?” (B5)

Students were interested in using the device in several contexts including during school, sports, and sleep. They also recommended features for the device so that its able to be helpful during the school day when they don't have their phone. Since the device doesn't have a screen, students recommended lights indicating different ranges of heart activity or as a breath pacer in addition to sensory buttons.

“When your heart rate is up a light comes up to help your breathing like a coloured blinking light” (C3)

“Put a fidget button on device” (C3)

“Green light when your heartbeat is steady” (C4)

### 3.14 Co-design evaluation

Following the co-design sessions, an evaluation survey was shared with students with the aims of (1) measuring how closely students felt the sessions aligned with PPI participant engagement values, and (2) confirming the main takeaways from the sessions with students.

In the survey, students ranked how well they felt the co-design aligned with the participatory engagement guidelines. All values were rated as “excellent” by over 50% of the student respondents with collaboration and equity and inclusion as the best performing values while flexibility had the most students indicating it as “needs improvement” (Table 6).

**Table 6:** Participatory engagement value evaluation (*n* = 51).

How well has each value been met during the co-design?	Excellent	Reasonable	Needs improvement
Respect—the important role of students is made clear	64%	34%	2%
Trust—everyone can voice an opinion	69%	29%	2%
Flexibility—the schedule of students is considered	55%	37%	8%
Transparency—project goals and session activities are clear	60%	36%	4%
Power Sharing—there is a variety of ways for students to contribute	68%	32%	0%
Collaboration—students are co-scientists in the project	72%	24%	4%
Equity &. Inclusion—barriers to engagement are minimal and all students’ voices are heard equally	74%	26%	0%

Forty-four students expressed overall satisfaction with the co-design process, while seven students noted areas where the participatory engagement values could be improved. Students were also given the opportunity to expand on their feedback of the co-design sessions. One student shared their engagement with the process:

“Overall, it was really enjoyable!! I would look forward to each of the sessions, and now that we’ve been granted the opportunity to actually trial it, I can’t wait! As someone who does suffer from stress, it would be great to have the resources to find the best way to work around anything I'm feeling”. (A)

#### 3.14.1 Co-design takeaways

Students ranked their interest in using different sections of a well-being app. Students (*n* = 62) rated their interest, on a scale from 1 to 5 (1 being not interested and 5 being very interested), in app tabs for resources (mean = 3.3, SD = 1.2), wearable data tracking (mean = 3.7, SD = 1.0), and chat-based human coaching (mean = 2.8, SD = 1.2). The human coaching tab was the only feature with a shift towards students not interested in this feature. Similarly, students confirmed their interest in positive app features that emerged during the co-design session. Students showed the greatest support for app customizability and organizational tools.

#### 3.14.2 Naming “wellby: your well-being buddy”

At the end of the form, students had the option to suggest a name for the app and wearable that will be developed based on this co-design. Suggested names were entered into a list for students across the schools to vote on. The winning name was chosen as “Wellby: Your Well-being Buddy”, suggested by a student at School A, which will be used to refer to both the app and wearable device co-created with students.

## 4 Discussion

### 4.1 Summary of findings

This study aimed to outline a co-design process with Irish secondary school students to explore their well-being needs and inform the development of student-centered digital tools. The outcomes revealed students' well-being goals and their design preferences, offering insights into how digital well-being interventions can be meaningfully integrated into students' daily life. These findings represents the initial stages of a co-creation process to develop “Wellby”, a mobile app and wearable device aimed at supporting Irish secondary school students' well-being. The outcomes of these co-design sessions set the stage for continued collaboration with students in the iterative development of Wellby. Involving students as equal partners throughout this process will help to ensure that the mobile app and wearable remain aligned with student needs and effectively address their well-being goals.

The findings from the needs assessment highlighted the importance of addressing student stress and sleep management, as these were consistently identified as top priorities by students, parents, and teachers. Students demonstrated a complex understanding of the impacts of stress and its interconnectedness with sleep, mental and physical health, social connections, and everyday behaviors. While many students already employ a range of stress management strategies, they expressed a clear need for additional tools to better support their well-being and an interest in using a student-centered well-being app and wearable. Based on the needs identified, there is alignment between the sources of stress in students, such as academic pressure and poor sleep, and the mechanism through which mobile apps can support them. App features such as biofeedback, mood tracking and educational resources can help support stress and well-being management by promoting self-regulation and awareness, based on self-regulation theory and well-being informed design (50, 53).

In addition to shared well-being needs around stress and sleep, the findings revealed important variations across student groups and school types. Student-centered tools must consider the range of well-being needs present in different student groups and be able to adapt to the unique needs of individual students. In this study, students at mainstream schools expressed a greater interest in support for exam-related stress management, while students at Youthreach centers more frequently highlighted environmental stressors and sought support in areas such as risky substance use and sleep. This is likely influenced by the structural differences of Youthreach centers compared to mainstream schools that are more exam focused. Interestingly, despite experiencing higher levels of drowsiness and fewer students meeting recommended sleep guidelines, Youthreach students expressed less desire for additional well-being support compared to their mainstream school counterparts. This may reflect the specialized and personalized supports already embedded within Youthreach centers or could suggest the presence of stigma around seeking additional help.

Demographic differences between the schools may also contribute to the variations in stress levels and support needs. Notably, School A, an all-female school, reported significantly higher subjective stress levels and a stronger interest in stress management support than the co-educational schools, consistent with existing research showing higher stress among female adolescents (54–56). These insights from the needs assessment provided a strong foundation for initiating the co-design process where students further shaped the features of Wellby.

During the co-design sessions, the diversity of student preferences that emerged underscores the importance of developing customizable and accessible tools rather than rigid, one-size-fits-all solutions. For example, while some students enthusiastically supported the idea of a daily positive quote on the app's home screen, others found it “cringey” and preferred not to have it. To address this, the Wellby app will include an optional daily quote feature that users can toggle on or off. Similarly, students will have the ability to customize the app's color scheme and choose strap colors and materials for the wearable device, ensuring the tools align with their individual preferences. The emphasis on customizable features in Wellby highlights the importance of allowing users to tailor tools to their individual strengths and preferences while also demonstrating the pride that students feel in getting to design features of Wellby themselves. This approach not only enhances user satisfaction but also aligns with PHS by fostering a sense of agency and control around student's lifestyle habits.

In addition to customizable features, the theme of digital well-being emerged during the co-design sessions which focused on students' relationship with technology as a critical component in the development of tools for adolescents. Students acknowledged the dual role of technology in their lives: while it can exacerbate stress through features like social media comparison, it also offers opportunities for connection, self-expression, and stress management. Teachers echoed this by sharing concerns about excessive reliance on phones while recognizing the potential benefits of thoughtfully designed digital tools. These findings underscore the importance of adopting a positive design approach by addressing potential risks, such as privacy, biased algorithms, and addictive features, while ensuring tools meet students' psychological needs, leverage the strengths of digital interventions, and encourage healthy technology use.

The need for positive technology became evident in the co-design sessions during discussions about artificial intelligence (AI). Students expressed both enthusiasm and concern around the integration of AI into well-being support tools. With increasing exposure to text-generating AI, many students suggested incorporating this feature in the Wellby app. Some viewed AI as an opportunity to offer low-pressure, private chat support and mentioned already using AI chatbots for this purpose. Others, however, expressed distrust in the accuracy of AI-generated information and discomfort engaging with a non-human chatbot. Students discussions showed awareness around valuable ethical considerations which align with similar research (57) and should be taken into account in the adoption of generative AI within digital well-being interventions for students.

Another area of interest expressed by students was self-tracking, particularly using wearable technologies. It is important to students that these devices are discrete, fashionable, and minimally distracting. Students suggested practical features for the wearable, such as light indicators to convey heart activity ranges or to guide breathing exercises, which could provide utility during school hours when phones are often unavailable.

While student well-being was the focus of this project, a need to support Irish teachers' well-being also emerged throughout this process, particularly in the Youthreach Centers. This aligns with broader findings from the 2022 Youthreach Employee well-being report, which highlighted systemic stressors affecting these educators (58). Expanding co-design efforts to include teachers and school staff could yield tools that address both student and teacher well-being, fostering a holistic approach to school health.

### 4.2 Design approach and methodological takeaways

The participatory design approach in this study reflects a commitment to treating students as co-researchers, aligning with principles from the PPI Ignite Framework, Radical Participatory Design (RPD), and the WHO (44). The RPD approach challenges traditional hierarchies by involving students not only in feedback but also in decision-making processes. Future iterations could further decentralize decision-making by enabling students to co-facilitate sessions, lead specific phases of design, and guide the study methodology itself.

Additionally, psychological and behavioral insights can help to mitigate risks and support psychological wellbeing in the development of digital well-being interventions. Frameworks from PHS and well-being informed design can help to incorporate support for both lifestyle habits and psychological needs into the design of well-being conscious technologies (47, 48, 50). This study methodology merges participatory design and PHS to emphasize the importance of not only aligning digital tools with the end-users needs but also examining the overall functionality and delivery of these tools to promote flourishing, a key aim of PHS.

This study aimed to bridge participatory design with PHS to co-create a digital tool to support student well-being. It began with a needs assessment that gauged interest in digital tools for well-being and confirmed the need for further exploration through co-design. A critical feature of this study was the sharing of findings with students at the end of each phase to gather their feedback and interpretation of the survey and workshop outcomes. Additionally, the use of diverse methods, including surveys, focus groups, and hands-on design activities, enabled more accessible ways for students to share feedback and provided a nuanced understanding of student needs and preferences (24).

The findings from this study also highlight the potential of adolescent-driven digital interventions to support resilience among this cohort. Ungar and colleagues have conducted extensive studies with adolescents with adverse childhood events in their past as well as those experiencing current trauma. They show that resilience is not solely an individual trait, but consists of both the individual “rugged qualities”, such as a growth mindset and healthy lifestyle behaviors, and access to supportive external resources (59). These resources can be provided by government agencies, schools, NGOs, or community networks. In this context, participatory design approaches that emphasize collaboration with students and teachers, such as the development of Wellby, may serve as an additional resource to help foster resilience among young people.

### 4.3 Challenges and limitations

Several challenges were encountered during this study. One major challenge was determining how to incorporate the diverse range of preferences and requests from students for app and wearable device features. Students were asked to vote for the features they would use most from the options that they suggested, and the most highly voted features were prioritized. However, some conflicting preferences and features had to be excluded due to design scope limitations. Another challenge was ensuring that students remained focused and engaged throughout the co-design sessions. While most participants engaged meaningfully in each session, it is also important to consider the influence of peers, distraction, and classroom fatigue during sessions, which at times made it challenging to discern the authenticity of answers reflecting student needs. Another limitation was the balance between student involvement and researcher guidance. While the co-design approach aims to empower students as co-creators, it is essential to ensure they are adequately informed about the goals and constraints of the project to contribute effectively. Providing additional resources and opportunities for students to ask questions at the outset could help students feel more prepared to engage meaningfully. Time constraints also posed challenges, particularly given the diversity of schools involved and the need to adapt the co-design process to fit each context. While the condensed session at School D provided valuable insights, it limited the depth of engagement compared to other schools.

Beyond these challenges encountered during implementation, the study also has broader limitations that should be considered. The generalizability of the co-design outcomes could be strengthened through a greater number of student participants at each school, as well as broader representation from different types of schools, such as private and Delivering Equality of Opportunity in Schools (DEIS) schools. Increased involvement of parents, legal guardians, and teachers could also enrich this study. Additionally, the participatory nature of this study could be expanded by giving students a stronger role in shaping the study methodology and guiding key phases of the design process. Future co-design studies could benefit from allocating more time for participant-led planning, including diverse participant cohorts, and providing more frequent opportunities for students to give feedback and shape the direction of the intervention.

### 4.4 Implications

The findings from this study contribute to the growing field of adolescent digital health by demonstrating how co-designed interventions can address the need for accessible health technologies that are adolescent-focused, scalable, and engaging (44, 60, 61). Students shared examples of mobile apps they currently use to support their well-being, which were predominantly social media platforms like YouTube and TikTok, rather than well-being focused tools. While these platforms can promote social connection and enhanced access to information, they are associated with numerous risks such as cyberbullying and declining mental health among youth (62, 63), highlighting the urgent need for healthier spaces for young people to spend their time online. Their expressed interest in student-centered digital well-being interventions echoes the gap for these types of interventions identified in the literature (15–17). Participatory engagement processes that allow students to guide the development of these interventions can help to ensure that they are relevant and meaningful for adolescents (28, 29). These tools must also account for ethical considerations around safety and accountability particularly related to emerging features such as generative AI (40, 57, 64).

While this study focused on Irish secondary school students, the participatory methods and design principles are applicable to diverse populations, offering some guidance on creating culturally and contextually relevant health interventions. Future work in the development of digital well-being interventions should encourage active, participant-led processes and consider well-being informed design practices to encourage the design of interventions and digital interfaces that promote the physical and mental flourishing of end-users (47, 48, 50).

## 5 Conclusion

This study demonstrates the potential of co-design to create digital tools that address key well-being needs of adolescents. It also highlights the need for the development of student-centered and customizable positive technology that is informed by well-being sciences alongside students' insights. By engaging students as co-researchers and incorporating their insights at every stage of development, Wellby represents a co-created mobile app and wearable to supporting the well-being of Irish secondary school students. The next phase will build on these findings through a feasibility study to further refine and evaluate Wellby with students to ensure it remains meaningful, usable, and aligned with students' needs.

## Data Availability

The datasets presented in this article are not readily available because The summary data presented in the paper including quotes and descriptive statistics are made available. Requests to access the datasets should be directed to Justin Laiti, justinlaiti22@rcsi.ie.
